# Monitoring expression of yeast cell wall protein-encoding genes in response to high hydrostatic pressure

**DOI:** 10.1186/1753-6561-8-S4-P205

**Published:** 2014-10-01

**Authors:** Tassia Nati, Fernanda Bravim, Jimmy Soares, Mainã Mantovanelli Mota, James Riley Broach, Antonio Alberto Ribeiro Fernandes, Patricia Machado Bueno Fernandes

**Affiliations:** 1 Núcleo de Biotecnologia, Universidade Federal do Espírito Santo, Vitória, Espírito Santo, Brazil; 2 Penn State University College of Medicine, Hershey, PA 17033, USA

## Background

The cell wall (CW) is one of the most important structures of the yeast cell, accounting for up to 30% of its dry weight. This organelle determines cellular morphology, affords mechanical protection and provides osmotic support. The yeast CW is a dynamic structure susceptible to many modifications, adjusting its composition and thickness to environmental changes. These responses usually involve changes in gene expression, increasing levels of proteins that have protective functions. High hydrostatic pressure (HHP) is a useful model of stress, which causes CW compression [1]. Exploring this process using the model organism *Saccharomyces cerevisiae *may allow us to understand the mechanisms of yeast stress tolerance in biotechnological processes and it may also helps in searching for effective antifungal drugs, since the CW is a desirable target of action.

## Methods

In this work *S. cerevisiae *strain BT0510 was subjected to a non-lethal HHP of 50 MPa for 30 min, followed by recovery at atmospheric pressure for up to 15 min. RNA samples were collected to perform a time series microarray expression analysis.

## Results and conclusions

Through bioinformatics, changes in the expression pattern (≥2 fold) of several CW organization and biogenesis genes were identified. HHP induced the expression of *HSP12*, which protein is present in CW and acts by increasing its flexibility [2], promoting survival under many stress conditions. The CW stress adaptive response is mainly mediated via Cell Wall Integrity (CWI) pathway, and its genes were affected by HHP. Rho1p is the master regulator of CWI signaling, and is stimulated by Rom1p [3]. HHP induced the expression of *ROM1*. Mtl1p and Wsc3p are related in detecting and signaling CW status to Rho1p; their genes were upregulated by HHP. Related to the same pathway, HHP activates *PKH1 *and *PKH2*, paralog genes of which proteins activate components of a signaling cascade required for CWI maintenance. The genes related to β-1,3-glucan, β-1,6-glucan and CW chitin biosynthesis were not strongly affected by HHP. Furthermore, *MNN1, MNN9 *and *MNN10*, correlated with protein mannosylation were downregulated by HHP. The products of these genes are subunits of mannose polymerase complexes, what suggest a possible change in the outer layer of the CW. Moreover, HHP induced the coding genes of Pir3p Hsp150p, members of proteins with internal repeats family (PIR), correlated with CW reinforcement by interconnecting two or more β-1,3-glucan molecules providing defense against β-1,3-glucanases, common stress in the wild since these enzymes abound in plant tissues [4]. *DSE2, DSE4, EGT2, CTS1, SCW11 *and *SUN4*, related with CW degradation and separation of daughter cell from the mother cell, were downregulated by HHP, suggesting that pressure can affect cell division. Many genes involved in CW biosynthesis and organization had their expression changed after HHP treatment, evidencing the importance of the CW to ensure cell survival against this stress. Knowing the key cell-survival proteins is critical to improve biotechnological processes, and the results presented here may help in development of new drugs or in develop stress tolerant distillery yeast cells.
